# The “*Journal of Functional Morphology and Kinesiology*” Journal Club Series: Highlights on Recent Papers in Exercise and Osteoarthritis

**DOI:** 10.3390/jfmk4010007

**Published:** 2019-01-17

**Authors:** Giuseppe Musumeci, Marta A. Szychlinska, Walter Herzog

**Affiliations:** 1 Department of Biomedical and Biotechnological Sciences, Human Anatomy and Histology Section, School of Medicine, University of Catania, Via S. Sofia 87, 95123 Catania, Italy; 2 Faculty of Kinesiology, Engineering, Medicine and Veterinary Medicine, University of Calgary, Calgary, AB T1X 0L3, Canada

## Abstract

We are glad to introduce the eleventh Journal Club. This edition is focused on several relevant studies published in the last years in the field of exercise and osteoarthritis, chosen by our Editorial Board members and their colleagues. We hope to stimulate your curiosity in this field and to share with you the passion for sport seen also from the scientific point of view. The Editorial Board members wish you an inspiring lecture.

## 1. Introduction

Osteoarthritis (OA) is a degenerative disease of the articular cartilage, and it represents one of the most common causes of disability in the world linked to hypomobility [1]. It leads to social, psychological, and economic costs with financial consequences. Different OA treatments are usually considered in relation to the stage of the disease, such as surgical management, pharmacological treatments, and non-pharmacological treatments such as physical activity [1]. In relation to mild OA, non-pharmacological and behavioural treatments are recommended because they are less invasive and better tolerated by patients. All of these treatments that are used to manage OA are problematic, but solutions to these problems are on the horizon. For this reason, we decided to realize this editorial because, until today, there has been very little information regarding the physical treatment of this important disease. There is limited evidence, in the current literature, for the higher benefits of one type of training over another in the treatment of mild OA. Exercise programs that combine strengthening exercises with exercises that increase flexibility and aerobic capacity seem to be the “best” option to offer to patients with mild OA, taking into account their preferences and tolerance. For this reason, we talk about adapted or tailor-made physical activity. 

## 2. Recent Papers Regarding Exercise and Osteoarthritis

### 2.1. Can Exercise Prevent Knee Osteoarthritis? A Possible Biological Key!

#### Highlight by Giuseppe Musumeci

The elegant article by Jennifer Abbasi [2] is important because it deals with the interesting and poorly analyzed topic of the beneficial effects of exercise in preventing and ameliorating osteoarthritic cartilage. Current evidence suggests that exercise is effective in enhancing musculoskeletal strength and power capacity, and improving physical and psychological conditions in patients with osteoarthritis, although the mechanisms mediating these effects are still not well understood [3]. I would respectfully add a possible biological key to explain the effects of physical activity and diet on cartilage, since our group has focused its attention on OA animal models for years. In particular, we have studied joint lubrication related to the presence of a glycoprotein called lubricin [4]. Since in OA cartilage molecular changes occur before structural changes, it would be reasonable to direct therapy towards the molecules participating in the early phases of the disease. For this reason, we should focus our attention on the mechanism of synoviocyte dysfunction during the OA process, which should be a candidate as a missing link. These cells can produce and secrete a number of enzymes and cytokines/chemokines that mediate tissue damage and inflammation. In pathological conditions, macrophages stimulate synoviocytes to produce matrix degrading enzymes, resulting in the OA process [5]. Joint immobilization inhibits the release of synovial fluid, which is rich in hyaluronan and lubricin, within the joint cavity, facilitating future damage and chondrocyte apoptosis [6]. Otherwise moderate physical exercise, normal joint loading, and mechanical stimulation, both in vivo and in vitro, improve lubrication and chondrogenesis [7], and prevent cartilage degeneration by promoting lubricin synthesis in synovial fluid. Moreover, a combination of a diet based on extra-virgin olive oil coupled with moderate physical activity has been shown to be protective against inflammation in rat articular cartilage after induced OA [8]. In other words, the quality of diet does matter. Adapted moderate physical activity could regulate this unknown mechanism leading to synoviocyte dysfunction upstream of early OA and consequently delay the onset of advanced OA. Potential physical and non-pharmacological treatments of initial stages of OA could postpone the need for joint replacement [9]. Exploring features of the therapeutic targets of physical exercise and a healthier diet may offer a new way of inhibiting the OA process, limiting the deleterious effects of chondrocyte senescence [10]. The taboo that joints affected by osteoarthritis should be used as little as possible should be absolutely disproved, as national and international guidelines, including EULAR and OARSI, currently recommend exercise in the management of OA.

### 2.2. Exercise and Osteoarthritis

#### Highlight by Walter Herzog

Osteoarthritis is a multi-factorial disease of joints. One factor that has been implicated in the onset and progression of OA is mechanical loading of joints, particularly the knee and hip joints in humans (OA is a wear and tear disease) [11]. Exercise invariably increases loading of the musculoskeletal system, including joints [12], and sports-related loading, for example in soccer players and weight lifters, has been identified as having an increased risk for the development of OA [13]. Nevertheless, moderate exercising is often promoted as a way to retain musculoskeletal health and prevent OA (e.g., in Roos et al.’ study [14]). Unfortunately, strong, systematic, and longitudinal studies on long-term exercising and OA development and progression in humans are rare, and opinions are controversial. 

Exercising strengthens muscles, and quadriceps muscle weakness has been identified as an independent risk factor for the onset of OA in rabbit knees [14,15]. Therefore, it appears appealing to conclude that strengthening muscles (particularly the quadriceps muscles in humans) prevents (knee) OA. However, compelling experimental evidence for this statement is missing. 

Obesity and associated increases in body weight have been implicated with lower limb joint loading that may lead to OA. However, obesity in humans does not only cause OA in joints loaded by body weight, like the knee, but also produces increased rates of OA in finger joints that are not loaded by body weight, suggesting that obesity-induced OA is not necessarily associated with mechanical over-loading (e.g., Grotle et al., 2008 [16]). Rather, low-level systemic and local inflammation in obese animals has been shown to trigger OA [17]. We demonstrated that a high-fat/high-sucrose (HFS) diet-induced obesity is a powerful initiator of knee OA in Sprague-Dawley rats [18]. Moderate exercise (running five times per week for 30 min) completely prevented knee OA in these HFS diet-exposed rats, not because of a reduction in body weight or body fat percentage, but by alleviating low-level local and systemic inflammation in these animals (Rios et al., unpublished observation). The exercising animals were still obese, but were metabolically healthy, which appears to be key in preventing knee OA.

In summary, quadriceps muscle weakness has been shown to be an independent risk factor for the onset of knee OA in rabbits [14,15] and possibly humans [19]. Maintaining quadriceps strength through moderate exercise might reduce the risk of knee OA. Obesity and associated metabolic syndromes cause knee OA in rats [17], and are associated with an increased occurrence of knee OA in humans. Moderate aerobic exercise (running) prevents knee OA in rats completely by establishing a healthy metabolic environment (Rios et al., unpublished observation), while the relationship between moderate aerobic exercise and obesity-induced OA remains unclear in humans.

### 2.3. Hamstring Muscle Activation During the Hip Extension and Nordic Hamstring Exercises and Injury Prevention Programs in Women

#### Highlight by Marta A. Szychlinska

There is evidence that muscle dysfunction is involved in the pathogenesis of knee osteoarthritis. The lower limb muscles represent the natural brace of the knee joint, and so important muscle dysfunction may arise from either quadriceps or hamstring weakness [20]. Understanding hamstring muscle activation patterns in resistance training exercises may have implications for the design of strength training and joint injury prevention programs. In a recent and interesting study by Messer et al., entitled “Hamstring Muscle Use in Women During Hip Extension and the Nordic Hamstring Exercise: A Functional Magnetic Resonance Imaging Study”, the spatial patterns of hamstring muscle activity during the 45° hip extension and Nordic hamstring exercises have been determined in women by using functional magnetic resonance imaging (fMRI) [21]. It is a cross-sectional study in which six recreationally active women with no history of lower-limb injury underwent fMRI on both thighs before and immediately after five sets of six bilateral eccentric contractions of the 45° hip extension exercise or the Nordic hamstring exercise. The latter is a form of eccentric exercise used to strengthen the hamstrings eccentrically [22]. Using fMRI, the transverse (T2) relaxation times were measured from pre-exercise and post-exercise scans, and the percentage increase in T2 was used as an index of muscle activation. The fMRI revealed a significantly higher biceps femoris long head-to-semitendinosus ratio during the 45° hip extension exercise than in the Nordic exercise. The T2 increase after the 45° hip extension exercise was greater for the biceps femoris long head, semitendinosus, and semimembranosus than that of the biceps femoris short head. During the Nordic exercise, the T2 increase of the semitendinosus was greater than that of the biceps femoris short head and biceps femoris long head. While both exercises involve high levels of semitendinosus activation in women, the Nordic exercise preferentially recruits this muscle, while the hip extension exercise more evenly activates all the biarticular hamstrings. These findings may have implications for the design of hamstring and joint injury prevention programs with the consequent prevention of osteoarthritis onset.
